# THE ROLE OF SPIRITUALITY IN LATER LIFE: A STUDY OF OLDER ADULT UNIVERSITY STUDENTS IN PORTUGAL

**DOI:** 10.1093/geroni/igac059.2431

**Published:** 2022-12-20

**Authors:** Giuliana Casanova

**Affiliations:** University of Aveiro/ University of Porto , Leca do Balio , Porto, Portugal

## Abstract

In Portugal, little has been done to investigate spirituality and its impact on later life. This study aims to discover whether spirituality contributes to successful aging in the lives of Portuguese older adults. This mixed-methods research study is based in Porto, Portugal. The sample size is 58 students from universities of the third age, ages 65 to 89 with no cognitive deficit. Data was collected by a demographic questionnaire, Spirituality Scale, Scale of Psychological Well-Being, and a mix method questionnaire. The data analysis was conducted by descriptive statistics and thematic analysis.

Results: Participants rated high in both the Spirituality Scale and the Psychological Well-Being Scale. The data also showed a significant and positive correlation between the dimension of hope/optimism from the Spirituality Scale and the dimension of autonomy from the PWB Scale; as well as between global spirituality and the dimension of autonomy. The mix-methods questionnaire showed 90% of the participants believe in God or a higher power, 81% considered themselves spiritual while 89.4% felt spirituality had always been a part of their lives. The three most significant themes that emerged were the connection to a higher power/divine, personal well-being, and the importance of spirituality throughout their lifetime. Discussion: Spirituality demonstrated to be correlated with the participant's ability and perception of autonomy and independence; having their spirituality as a coping mechanism to deal with life′s adversities while being able to look at the future with optimism. Spirituality can provide purpose, guidance, and meaning in the later years of life.

